# Oseltamivir phosphate for suspension is bioequivalent to TAMIFLU in healthy volunteers: a randomized, open-label clinical study

**DOI:** 10.1186/s40360-023-00646-1

**Published:** 2023-02-21

**Authors:** Ying Wang, Bangzhong Tang, Jing Xie, Xiaoqin Wang, Peng Ren, Guangmei Wu, Cuixia He, Minhui Zhu, Yue Su, Jiaxiang Ding, Yuanyuan Xu, Ling Fan, Qin Ge, Yuzhou Ding, Juan Zhu, Bingyan Liu, Rongfang Shan, Huan Zhou

**Affiliations:** 1 National Institute of Clinical Drug Trials, The First Affiliated Hospital of Bengbu Medical College, Bengbu, Anhui China; 2 School of Pharmacy, Bengbu Medical College, Bengbu, Anhui China; 3 Shenzhen Beimei Pharmaceutical Co, Ltd, Shenzhen, Guangdong China; 4 School of Public Foundation, Bengbu Medical College, Bengbu, Anhui China

**Keywords:** Influenza, Oseltamivir Phosphate for suspension, TAMIFLU®, Bioequivalence, Pharmacokinetics, Safety

## Abstract

**Purpose:**

The study was aimed at evaluating the bioequivalence and safety of oseltamivir phosphate for suspension, provided by Shenzhen Beimei Pharmaceutical Co. Ltd. and manufactured by Hetero Labs Limited, and the reference product TAMIFLU® in healthy Chinese subjects.

**Methods:**

A single-dose, randomized, two-phase, self-crossed model was adopted. Among 80 healthy subjects, 40 subjects in the fasting group and 40 subjects in the fed group. Subjects in the fasting group were randomized into two sequences according to the proportion of 1:1, each given 75 mg/12.5 mL of Oseltamivir Phosphate for Suspension or TAMIFLU®, and cross-administered after 7 days. Postprandial group is the same as fasting group.

**Results:**

The T_max_ of TAMIFLU® and Oseltamivir Phosphate for Suspension in the fasting group were 1.50 h and 1.25 h, which in the fed group were both 1.25 h. Geometrically adjusted mean ratios of the PK parameters of Oseltamivir Phosphate for Suspension along with TAMIFLU® under fasting and postprandial conditions were in the range of 80.00–125.00% at the 90% confidence interval (CI). The 90% CI of C_max_, AUC_0-t_, AUC_0-∞_ for fasting group and postprandial group were (92.39,106.50), (94.26,100.67), (94.32,100.89) and (93.61,105.83),(95.64,100.19),(96.06,102.66). Among the subjects on medication, a total of 18 subjects reported 27 adverse events, all of which were treatment-emergent adverse events (TEAEs), six of these TEAEs were rated as grade 2 in severity and the rest were as grade 1. The number of TEAEs in the test product and the reference product were 14,13 respectively.

**Conclusion:**

Two Oseltamivir phosphate for suspensions are safe and bioequivalent.

**Supplementary Information:**

The online version contains supplementary material available at 10.1186/s40360-023-00646-1.

## Introduction

Influenza, a contagious respiratory disease, which induced by the influenza virus that resulting in high morbidity and mortality worldwide. Outbreaks of influenza are usually earliest detected in children with fever illness [1]. The inflation on hospitalizations and the death by or from respiratory and circulatory disease due to influenza has been disclosed as the epidemiology proceeds. The typical symptoms of influenza usually peak 2–3 days after the onset and sustains until 4–5 days. According to WHO estimates, globally, influenza epidemics cause about 3–5 million severe cases and about 290,000–650,000 deaths related to respiratory diseases each year [2]. As approved by the US FDA, Anti influenza-virus drugs shall be given within 48 h of the occurrence of influenza symptoms. However, studies [3, 4] have confirmed that medication within 24 h of the outset of symptoms can achieve the best clinical benefit. When the duration of the disease is shortened by 24 h, its severity will be reduced accordingly, and the risk of serious complications of the disease will also be reduced accordingly.

Currently, four classes of antiviral drugs are approved for influenza treatment in several countries, mainly including adamantane, neuraminidase inhibitors, membrane fusion inhibitors, and RNA-dependent RNA polymerase inhibitors [5]. Among them, neuraminidase inhibitors are widely used. Neuraminidases are mainly distributed on the surface of influenza A and B viruses, and neuraminidase inhibitors bind competitively to them, making the sialic acid residues of the formed influenza virus particles unable to be cleaved, and the resulting process newly formed viruses cannot be released from the host cell after infection [6]. In contrast to adamantane, it is highly unlikely that drug resistance will develop [7].

Oseltamivir carboxylate, which is a neuraminidase inhibitor (NAI), is produced by hydrolysis of oseltamivir through the ester bond and inhibits the isolation of mature influenza virus from host cells by inhibiting neuraminidase, thereby inhibiting virus transmission in humans [8, 9]. Oseltamivir is an orally administered antiviral drug for the treatment and prevention of influenza A and B in adults and children over 2 weeks of age, which is a pre-drug of ethyl ester. It is usually well tolerated and side effects are rare. The most common adverse reactions are transient nausea and vomiting [2].

TAMIFLU® was jointly developed by Roche Registration Ltd and Gilead Sciences, Inc. The capsule was first approved for marketing by the FDA in October 1999, and the oral suspension was approved for marketing by the US FDA in December 2000. It has been marketed in many countries and regions around the world, and is recognized as one of the most effective drugs against influenza in the world. The purpose of this study was to assess the bioequivalence of oseltamivir phosphate for suspension (Hetero Labs Limited, 6 mg/ml after reconstitution, dose: 75 mg/12.5 mL) and TAMIFLU® (F. Hoffmann-La Roche AG, strength 6 mg/mL, dose: 75 mg/12.5 mL) in Healthy Chinese volunteers.

## Subjects and methods

### Study Design

This study was developed in healthy subjects in the fasting or postprandial state, using a single-dose, randomized, open-label, two-period, two-sequence, self-crossover design. It authorized by the Ethics Committee of the First Affiliated Hospital of Bengbu Medical College. The ethical approval process complies with the requirements of Good Clinical Practice (GCP), the Declaration of Helsinki and relevant Chinese laws and regulations. Registration number CTR20200764 at 26-Apr-2020. http://www.chinadrugtrials.org.cn/clinicaltrials.searchlist.dhtml.

According to "the Guiding Principles for Human Bioavailability and Bioequivalence test of Pharmaceutical Preparations (2015 Edition)", "the Guiding Principles for Human Bioequivalence Research of Chemical Drug Imitations with Pharmacokinetic Parameters as the End Point Evaluation Indicators", and recommended dose in Tamiflu® instructions, the single oral dose of 75 mg/12.5 mL was determined for the reference preparation and test preparation of this study. According to the data in the relevant literature [10], the intra-individual coefficient of variation (%CV) C_max_ of oseltamivir was in the range of 21.16–31.87%, and the AUC was in the range of 7.24–7.35%. Assuming one-sided α = 0.05, β = 0.2, %CV of 31.87%, a mean ratio F between the test product and reference product of 1, and the bioequivalence interval of 80.00–125.00%, the sample size of the two-period, crossover study design is 35 cases, calculated by PASS software. Considering the results of subject dropout and sample size estimation, 40 subjects were planned to be enrolled in this fasting trial and fed trial respectively, and the subjects' random numbers are K001-K040 and C001-C040 respectively. Screening examinations are performed in healthy subjects from 14 days prior to dosing to 2 days prior to dosing. Eligible healthy subjects were randomized in a 1:1 ratio into two groups for a single dose of 75 mg/12.5 mL of test and/or reference product, followed by a 7-day post-wash period and then crossover dosing. Subjects in the postprandial group had a high-fat breakfast calorie setting of 800–1000 kcal, with about 50% fat calories, 150 kcal of protein, and about 250 kcal of carbohydrates. The main food composition was milk, toast, peanuts, butter, sausage, and lettuce. Each high-fat meal was accurately weighed after a developed meal recipe. The interval time between the start of the high-fat meal and the start of dosing was 30 min, and all subjects finished the meal within 30 min. The sampling points are designed as follows: 0 h before administration (within 1 h before administration) and 5 min, 15 min, 30 min, 45 min, 1 h, 1.25 h, 1.5 h, 1.75 h, 2 h, 2.5 h, 3 h, 3.5 h, 4 h, 4.5 h, 5 h, 6 h, 8 h, 10 h, 12 h, 14 h, 24 h, 36 h, and 48 h after administration. The concentrations of oseltamivir and oseltamivir carboxylate in human plasma are established by liquid chromatography-mass spectrometry (HPLC–MS/MS). The quantitative linear range of oseltamivir is 0.500–200 ng/mL, and the LLOQ is 0.500 ng/mL. The quantitative linear range of oseltamivir carboxylate is 4.00–800 ng/mL, and the LLOQ is 4.00 ng/mL.

### Study participants

Subjects participating in the study: all aged between 18 and 65 years old (including the boundary values), with an appropriate gender ratio of males and females; men and women need to meet the standard weight of not less than 50 kg and 45 kg respectively, as well body mass index (BMI) ranged from 19.0–26.0 kg/m^2^ (both boundary values were included); subjects had no history of chronic or serious cardiovascular, such as hepatobiliary, renal, hematological and lymphatic, immunological, central nervous, neurological, gastrointestinal system diseases, etc., likewise health condition is well.

Subjects with the following situations need to be excluded: women who are breastfeeding, or subjects and their partners who are planning to become pregnant or donating sperm or eggs, unwilling or unable to voluntarily use effective contraception from the screening date to 6 months after the end of the trial; or subjects with history of psychiatric or neurological related diseases such as epilepsy or depression or family history of genetic diseases; or subjects taking any drugs or food supplements or herbal medicines within 14 days before screening or not longer than their 5 times half-life.

### Assessment and analysis

#### Pharmacokinetics

Pharmacokinetic parameters of oseltamivir and oseltamivir carboxylate were calculated using Phoenix WinNonlin (Pharsight Corporation, 8.3.1) based on individual blood concentrations and sampling time (calculated by actual sampling time) using non-compartmental analysis (NCA), C_max_, T_max_, AUC_0-t_, AUC_0-∞_, t_1/2_, λz, as well as %AUC_ex_ for oseltamivir and oseltamivir carboxylate were included. The bioequivalence analysis of two oseltamivir phosphate for suspension using C_max_, AUC_0-t_ and AUC_0-∞_ of oseltamivir in addition to oseltamivir carboxylate after natural logarithm transformation, was carried out by SAS® 9.4 software. If the 90% confidence interval (CI) of the ratio of the geometric mean of the pharmacokinetic parameters of the two products is within the range of 80.00–125.00% (including the boundary values), the test product is considered bioequivalent to the reference product. Tabular and descriptive statistical analyses were performed on subject distribution, demographic and other baseline characteristics, and other PK parameters.

#### Safety

Adverse events (AEs), serious adverse events (SAEs), concomitant medications, clinical laboratory tests, vital signs, 12-lead electrocardiogram and physical examination were recorded.

## Results

### Participants characteristics and baseline

A total of 205 subjects were screened, of which 125 failed in screening for not meeting the inclusion criteria/for meeting exclusion criteria. Among the 80 qualified subjects, there were 40 in the fasting group and 40 in the fed group. In the fed trial, 2 subjects withdrew early. Among them, Subject S107 (C001) voluntarily withdrew early from the trial during the washout period after completing the first period of blood sample collection; and subject S152 (C021) voluntarily withdrew early from the trial 4.5 h after the first period of dosing. In the fasting trial, one subject voluntarily withdrew early. Subject S082 (K033) withdrew early from the trial due to an adverse event of severe vomiting with high volume, which occurred 1.75 h after the second period of dosing. The baseline characteristics of the subjects are shown in Table 1.

**Table 1:** Summary of demography and its baseline characteristics

	Fasting group(*N* = 40)	Feding group(*N* = 40)
**Age(year)**	28.4(7.47)	29.9(8.82)
**Sex(n)%**		
Male	34(85%)	33(82.5%)
Female	6(15%)	7(17.5%)
**Height(cm)**	168.39 (6.454)	168.41(7.207)
**Weight(kg)**	64.12(7.027)	63.83(8.474)
**Body Mass Index (kg/m**^**2**^**)**	22.61(2.039)	22.46(2.168)

Data are Mean ± SD

### Pharmacokinetics

**Fig. 1:**
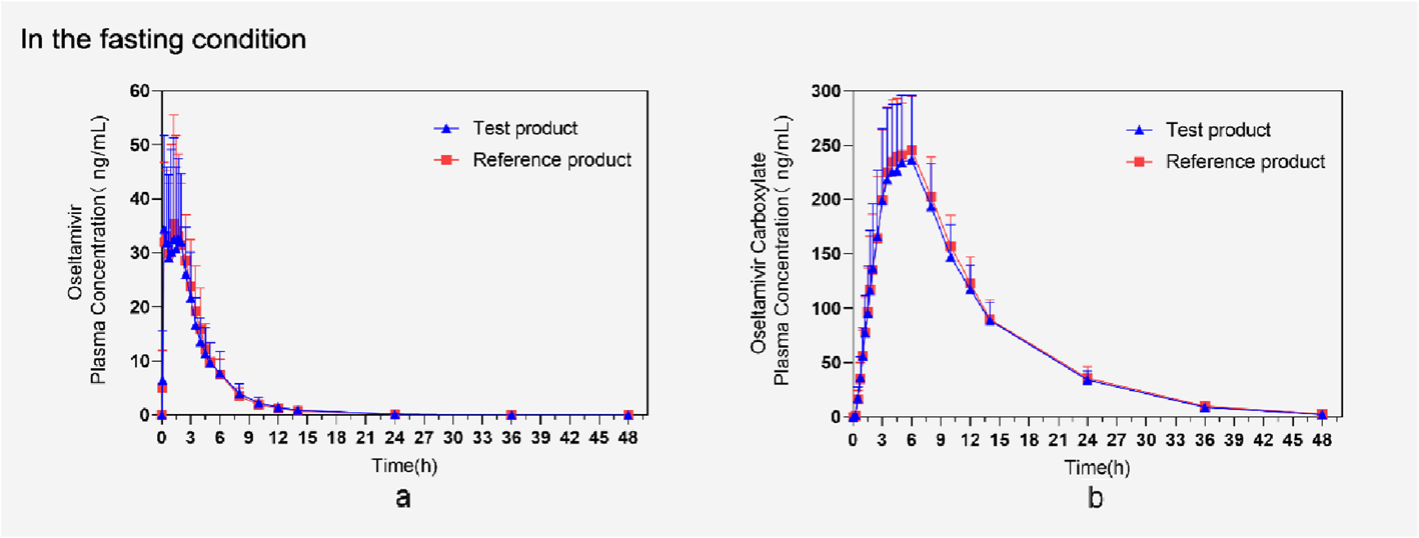
Mean (mean ± SD) plasma concentration vs. time profiles of oseltamivir and oseltamivir carboxylic under fasting conditions (pharmacokinetic concentration sets). **a** Mean (mean ± SD) plasma concentration–time profiles of oseltamivir after oral administration of test product (T) and reference product (R) in 40 subjects. **b** Mean (mean ± SD) plasma concentration–time profiles of oseltamivir carboxylic after oral administration of test product (T) and reference product (R) in 40 subjects. Subjects in the RT sequence group S048 (K018) were excluded from the pharmacokinetic analysis due to the occurrence of vomiting within twice the median time of T_max_ after administration of the first cycle, and none of the oseltamivir concentration data for that cycle

**Fig. 2:**
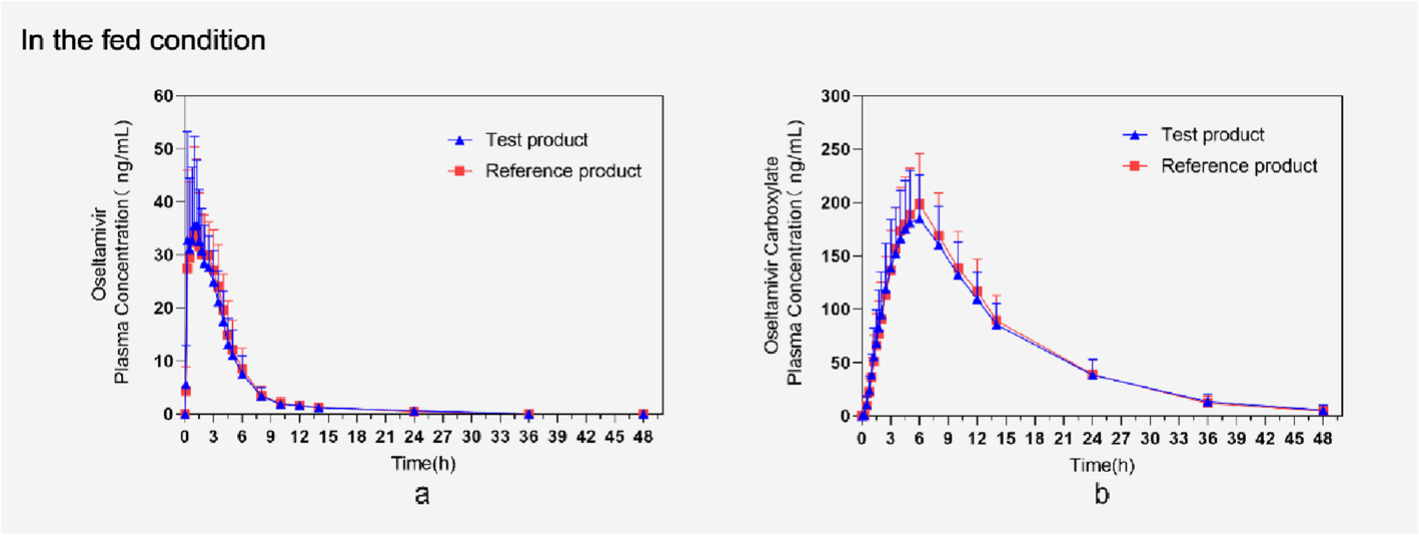
Mean (mean ± SD) plasma concentration vs. time profiles of oseltamivir and oseltamivir carboxylic underfed conditions (pharmacokinetic concentration sets). **a** Mean (mean ± SD) plasma concentration time profile of oseltamivir after oral administration of test product (T) and reference product (R) in 40 subjects. **b** Mean (mean ± SD) plasma concentration time profile of oseltamivir carboxylic after oral administration of test product (T) and reference product (R) in 40 subjects. Notes: T = test product of Oseltamivir Phosphate for Suspension; R = reference product of Oseltamivir Phosphate for Suspension(TAMIFLU®)

The mean oseltamivir and oseltamivir carboxylate in the fasting and fed groups had similar plasma concentration–time profiles (Figs. 1 and 2).

The pharmacokinetic parameters of oseltamivir and oseltamivir carboxylate in fasting or fed administration subjects included in the pharmacokinetic parameter analysis are shown in Table 2 and 3, T_max_ of oseltamivir in the fasting group was 1.50 h and 1.25 h, with C_max_ of 49.41 ng/mL and 48.79 ng/mL for the reference product and test product, respectively; T_max_ of oseltamivir carboxylate was both 5.00 h, with C_max_ of 257.95 ng/mL and 247.69 ng/mL, respectively. The T_max_ of oseltamivir of reference product and test product in the fed group were both 1.25 h, and C_max_ was 43.86 ng/mL and 44.16 ng/mL, respectively; the T_max_ of oseltamivir carboxylate was 6.00 h and 5.00 h, respectively, and C_max_ was 204.64 ng/mL and 194.13 ng/mL, respectively. The 90% CIs for the geometrically adjusted mean ratios of the PK parameters (C_max_, AUC_0-t_, AUC_0-∞_) of the test product and the reference product of oseltamivir phosphate for oral suspension in the fasting group were (92.39–106.50) %, (94.26–100.67) %, and (94.32–100.89) %, respectively. The 90% CIs for the geometrically adjusted mean ratios of the PK parameters (C_max_, AUC_0-t_, AUC_0-∞_) of the test product and the reference product of oseltamivir phosphate for suspension in the fed group were (93.61–105.83) %, (95.64–100.19) %, and (96.06–102.66) %, respectively.

**Table 2:** Statistical results of pharmacokinetic parameters of oseltamivir

	Fasting group		Fed group	
Parameter (unit)	Mean ± SD(%CV) (*N*)		Mean ± SD(%CV) (*N*)	
	Reference product	Test product	Reference product	Test product
*T_max_ (h)	1.50(0.25,3.50) (39)	1.25(0.25,3.00) (39)	1.25(0.25,4.00) (39)	1.25(0.25,4.00) (39)
C_max_ (ng/mL)	49.4051 ± 16.9687(34.35) (39)	48.7872 ± 17.7706(36.42) (39)	43.8615 ± 14.8930(33.95) (39)	44.1590 ± 16.9627(38.41) (39)
AUC_0-t_(h*ng/mL)	151.8912 ± 31.3853(20.66) (39)	147.8547 ± 33.8070(22.87) (39)	164.6864 ± 37.1978(22.59) (39)	160.5601 ± 34.3109(21.37) (38)
AUC_0-∞_(h*ng/mL)	156.0173 ± 32.0316(20.53) (39)	152.2639 ± 34.8439(22.88) (39)	172.0658 ± 39.0278(22.68) (39)	169.8901 ± 35.6103(20.96) (38)
%AUC_ex_(%)	2.66 ± 1.72(64.89) (39)	2.89 ± 1.53(52.88) (39)	4.16 ± 3.02(72.68) (39)	5.41 ± 5.08(93.96) (38)
λ_z_(1/h)	0.2716 ± 0.1287(47.38) (39)	0.2436 ± 0.1115(45.76) (39)	0.1884 ± 0.1219(64.72) (39)	0.1735 ± 0.1158(66.74) (38)
t_1/2_ (h)	3.34 ± 2.20(65.81) (39)	3.67 ± 2.15(58.52) (39)	6.18 ± 5.22(84.47) (39)	7.23 ± 6.41(88.69) (38)
CL/F(L/h)	502.6043 ± 113.2300(22.53) (39)	517.4635 ± 115.6803(22.36) (39)	456.4798 ± 97.2214(21.30) (39)	458.7121 ± 87.8661(19.15) (38)
V_d_/F(L)	2340.3764 ± 1432.7326(61.22) (39)	2628.4024 ± 1328.3567(50.54) (39)	3804.2724 ± 2984.5745(78.45) (39)	4627.3801 ± 4130.8730(89.27) (38)

T_max_ Time to reach maximum concentration, CV(%) Coefficient of variation, Intraindividual variation, C_max_ Maximum plasma concentration, AUC_0-t_ Area under the plasma concentration–time curve from time 0 to last time Concentration, AUC_0-∞_ Area under the plasma concentration–time curve from time zero extrapolated to infinite time, %AUC_ex_ Percentage of AUC0-∞ obtained by extrapolation, λ_z_ Elimination rate constant, t_1/2_ Elimination halflife, CL/F Apparent clearance, V_d_/F Apparent volume of distribution, Data are Mean ± SD, except *T_max_ represent the median (minimum–maximum)

**Table 3:** Statistical results of pharmacokinetic parameters of oseltamivir carboxylate

	The fasting group		The fed group	
Parameter (unit)	Mean ± SD(%CV) (*N*)		Mean ± SD(%CV) (*N*)	
	Reference product	Test product	Reference product	Test product
*T_max_ (h)	5.00(3.00,8.00) (39)	5.00 (3.00,6.00) (39)	6.00(3.50,8.01) (39)	5.00(3.50,8.01) (38)
C_max_ (ng/mL)	257.949 ± 53.7058(20.82) (39)	247.692 ± 63.4131(25.60) (39)	204.641 ± 45.5264(22.25) (39)	194.132 ± 47.4166(24.42) (38)
AUC_0-t_(h*ng/mL)	3159.4605 ± 537.1612 (17.00) (39)	3039.8997 ± 554.6876(18.25) (39)	2807.6025 ± 564.1152(20.09) (39)	2743.1544 ± 565.2824(20.61) (38)
AUC_0-∞_(h*ng/mL)	3229.8289 ± 543.0014 (16.81) (39)	3106.7115 ± 551.9343(17.77) (39) (18.25) (39)	2897.0655 ± 587.1272(20.27) (39)	2849.5667 ± 607.9339(21.33) (38)
%AUC_ex_ (%)	2.22 ± 0.72(32.52) (39)	2.23 ± 0.96(43.23) (39)	3.04 ± 2.15(70.62) (39)	3.63 ± 1.82(50.04) (38)
λ_z_(1/h)	0.1010 ± 0.0163(16.18) (39)	0.1032 ± 0.0146(14.18) (39)	0.0930 ± 0.0198(21.26) (39)	0.0878 ± 0.0191(21.70) (38)
t_1/2_ (h)	7.05 ± 1.18(16.70) (39)	6.85 ± 0.98(14.32) (39)	7.88 ± 2.21(28.04) (39)	8.28 ± 1.91(23.09) (38)

T_max_ Time to reach maximum concentration, CV(%) Coefficient of variation, Intraindividual variation, C_max_ Maximum plasma concentration, AUC_0-t_ Area under the plasma concentration–time curve from time 0 to last time Concentration, AUC_0-∞_ Area under the plasma concentration–time curve from time zero extrapolated to infinite time, %AUC_ex_ Percentage of AUC_0-∞_ obtained by extrapolation, λ_z_ Elimination rate constant, t_1/2_ Elimination halflife, Data are Mean ± SD, except *T_max_ represent the median (minimum–maximum)

All were within the range of 80.00–125.00%, and the conclusion of bioequivalence was established (Additional file 1). Subject S048 (K018) experienced vomiting within 2 times the median T_max_ of the same group of subjects after the first period of dosing, which may have an effect on PK parameters. Therefore, sensitivity analysis was performed on the bioequivalence of oseltamivir and oseltamivir carboxylate based on the original bioequivalence analysis without excluding subject S048 (K018). The results of the sensitivity analysis showed that the 90% confidence intervals for the geometrically adjusted mean ratios of the PK parameters (C_max_, AUC_0-t_, AUC_0-∞_) of the test product and the reference product of oseltamivir phosphate for suspension were all within the range of 80.00–125.00%, which was consistent with the conclusion of the bioequivalence analysis (Table 4).

**Table 4:** Statistical results of fasting oseltamivir pharmacokinetic parameters—sensitivity analysis

PK parameters (unit)	GLSM and ratio			CV (%)	90%CI	Power (%)
	Test product	Reference product	T/R(%)			
	(*N*)	(*N*)				
C_max_ (ng/mL)	45.9571 (39)	45.7018 (40)	100.56	18.58	(93.73,107.88)	> 99.9
AUC_0-t_ (h*ng/mL)	144.6536 (39)	146.6111 (40)	98.66	10.09	(94.95,102.53)	> 99.9
AUC_0-∞_ (h*ng/mL)	148.9203 (39)	150.6285 (40)	98.87	10.45	(95.01,102.88)	> 99.9

CV (%) Intraindividual variation, Coefficient of variation, C_max_ Maximum plasma concentration, AUC_0-t_ Area under the plasma concentration–time curve from time 0 to last time Concentration, AUC_0-∞_ Area under the plasma concentration–time curve from time zero extrapolated to infinite time, GLSM Geometric least squares mean

### Safety

Among the 40 subjects in the fed trial, a total of 9 subjects reported 14 adverse events, all of which were TEAEs, including elevated blood triglycerides, positive bacterial test (urine), positive urinary leukocyte esterase, urine sediment detection, abnormal urinalysis, elevated gamma-glutamyl transferase, elevated alanine aminotransferase, increased human chorionic gonadotropin, abnormal electrocardiogram QRS wave complex, and nausea. Of which 3 cases in 3 subjects were grade 2 in severity and the rest were grade 1 in severity. Adverse events of severity grade 2 are mainly elevated blood triglycerides. Vital signs and physical examination were all normal and no clinically significant. For the 12-lead ECG, 1 subject showed abnormalities with clinical significance; for laboratory tests, 4 subjects showed abnormalities with clinical significance after taking the drug for blood biochemistry tests, 3 subjects showed abnormalities with clinical significance after taking the drug for routine urine tests, 1 subject showed abnormalities with clinical significance after taking the drug for blood pregnancy tests, and no abnormalities with clinical significance after taking the drug for routine blood and coagulation tests. There were no abnormalities and clinically significant blood tests. Of the 27 cases of TEAEs in this trial, 14 were cured, 12 had an unknown outcome, 1 had a persistent outcome, and there were no serious adverse events (Additional file 2).

Subject with a positive pregnancy test (screening number S117, random number: C004), who had normal pregnancy test during the screening period and two-period enrollment examination, was suspected to be pregnant based on human chorionic gonadotropin (β-HCG) of 435.62 IU/L during the second-period exit examination on July 01, 2020, and was asked whether sexual intercourse has occurred, the subject explained that she once had sexual intercourse and took contraception before enrollment. The β-HCG doubled two days later and pregnancy was highly suspected. Pregnancy was confirmed by gynecological color Doppler ultrasonography, and the pregnancy event was reported and followed up. On March 12, 2021, the subject delivered a female infant at 39-week gestation, and the newborn was born naturally in good condition with an Apgar score of 10.

The more frequently reported adverse events after subjects took the test product and the reference product, ranked by system organ classification (SOC), were mainly elevated blood triglycerides in various tests, positive bacterial tests and positive urinary leukocyte esterase. The main gastrointestinal adverse event was nausea (Additional file 3).

Among the 40 subjects in the fasting trial, a total of 9 subjects reported 13 adverse events, all of which were treatment-emergent adverse events (TEAEs), this includes elevated C-reactive protein, decreased mean cell volume, elevated neutrophil count, nausea, and, as in the postprandial group, also elevated blood triglycerides, abnormal urinalysis, and vomiting. Of which 3 cases in 3 subjects were grade 2 in severity and the rest were grade 1 in severity. Vital signs, physical examination, and 12-lead electrocardiogram were all normal and no clinically significant; in terms of laboratory tests, after medication, 6 subjects showed abnormal and clinically significant blood biochemical tests, 2 subjects showed abnormal and clinically significant routine blood tests, 2 subjects showed abnormal and clinically significant routine urine tests, and pregnancy tests and coagulation tests were normal and clinically significant. Of the 13 cases of TEAEs in this trial, 8 were cured, 5 had an unknown outcome, and there were no serious adverse events (Additional file 2).

The more frequently reported adverse events after subjects took the test product and the reference product, ranked by SOC, were mainly elevated blood triglycerides in various tests, abnormal urinalysis, and elevated alanine aminotransferase. Gastrointestinal adverse events included vomiting and nausea (Additional file 3).

## Discussion

Oseltamivir phosphate, an antiviral drug, which used in the treatment of influenza A and B, and early treatment with oseltamivir was associated with a significant 33% reduction in ICU mortality compared with late treatment. It may be related to enhanced survival in critically ill patients with influenza pneumonia, at the same time, it may decrease the ICU length of stay and duration of mechanical ventilation [11]. Either when combined use of oseltamivir, lopinavir and ritonavir, was valid against the SARS CoV-2 protease, these drugs can be further explored for successful inhibition of COVID-19 for drug repurposing [12]. While a study of drug-pregnancy pharmacokinetic interactions in rhesus monkeys found that pregnancy had little effect on the pharmacokinetic parameters of oseltamivir and oseltamivir carboxylate [13], and oseltamivir had good safety data during pregnancy and was recommended by the CDC as a first-line treatment for pregnant women [14]. What we didn't expect was, one pregnancy case in the fed group occurred in our study. We followed the subject throughout her pregnancy, and found that the newborn was born in good condition. In addition, studies have also found that oseltamivir plays a role in affecting platelet function, and oseltamivir can be used as a promising therapy for adjuvant treatment of patients with reduced or defective thrombosis [15]. When combination of oseltamivir with dexamethasone may serve as a promising therapy for newly diagnosed primary immune thrombocytopenia, possibly due to the ability of oseltamivir to protect platelets from dealkylation and subsequent phagocytosis [16]. All in all, these are strong evidence of the value of clinical research and application of oseltamivir phosphate.

In this study, the oseltamivir phosphate for suspension produced by Hetero Labs Limited was compared with the originator TAMIFLU® to evaluate the absorption rate and degree of absorption in healthy humans, and to investigate the human bioequivalence of the two drugs. The difference between the test product and the reference product TAMIFLU® under fasting and fed conditions in the pharmacokinetic parameters between was not significant, which was similar to the results of previous studies [17, 18]. The T_max_ of oseltamivir of reference product and test product in the fasting group were 1.50 h and 1.25 h, respectively, and t_1/2_ were 3.34 h (65.81% CV) and 3.67 h (58.52% CV), respectively; the T_max_ of oseltamivir carboxylate were both 5.00 h, and t_1/2_ were 7.05 h (16.70% CV) and 6.85 h (14.31% CV), respectively. The T_max_ of oseltamivir of reference product and test product in the fed group were both 1.25 h, and t_1/2_ were 6.18 h (88.47% CV) and 7.23 h (88.69% CV), respectively; the T_max_ of oseltamivir carboxylate were 6.00 h and 5.00 h, respectively, and t_1/2_ were 7.88 h (28.04% CV) and 8.28 h (23.09% CV), respectively. The 90% CIs for the geometrically adjusted mean ratios of C_max_, AUC_0-t_, and AUC_0-∞_ of oseltamivir phosphate under fasting and fed conditions were (92.39–106.50)%, (94.26–100.67)%, (94.32–100.89)%, and (93.61–105.83)%, (95.64–100.19)%, (96.06–102.66)%, respectively, all within the range of 80.00–125.00%, which met the criteria for determining bioequivalence, and both were bioequivalent when administered under fasting and fed conditions. Based on the pharmacokinetic results after feeding and fasting states, we can also see that food causes changes in the values of the pharmacokinetic parameters of oseltamivir and oseltamivir carboxylate, where the AUC_0-∞_ of oseltamivir increases after feeding and the AUC_0-∞_ of oseltamivir carboxylate decreases, but this change has no effect on the clinical effect of oseltamivir. The C_max_, AUC_0-t_ of oseltamivir carboxylic acid were lowered under fed conditions, and some literature suggests that this may be related to an interaction with the carboxylesterase enzyme responsible for prodrug conversion [19].

This study confirmed the interchangeability of the test product and TAMIFLU® by verifying their bioequivalence, and that food intake did not affect the pharmacokinetics and bioequivalence of the two products. For patients, it is possible to achieve the same therapeutic effect by choosing a drug with a relatively good price.

In terms of adverse reactions, the most common side effects of oseltamivir phosphate were nausea (incidence 10%), vomiting (2–15%), abdominal pain, diarrhea, headache, insomnia and dizziness. Other side effects included conjunctivitis, rhinorrhagia, allergy, cardiac arrhythmias, gastrointestinal bleeding, erythema multiforme, toxic epidermal necrolysis, confusion, hepatitis, epilepsy, and neuropsychiatric events, but the incidence was < 1%. Oseltamivir is generally well tolerated [20–22]. In this study, adverse events such as nausea, vomiting, and elevated alanine aminotransferase were mild in severity and were mainly treated by observation.

The limitation of this study is as follows, the study is a bioequivalence trial in healthy Chinese subjects, the pharmacokinetic parameter data express the absorption distribution and elimination in Chinese subjects, and the bioequivalence in other races has not been evaluated yet, so it is not known whether there are differences between different races, which will be further verified in later trials.

## Conclusions

This study showed that oseltamivir phosphate for suspension (6 mg/ml after reconstitution, 75 mg/12.5 ml) and TAMIFLU® (6 mg/mL, 75 mg/12.5 ml) met bioequivalence criteria in the matter of the absorption rate and the degree of absorption of oseltamivir phosphate and oseltamivir carboxylate, and had good safety. Food had no effect on the pharmacokinetics and bioequivalence of oseltamivir phosphate for suspension and TAMIFLU® in healthy subjects.

## Supplementary Information

**Additional file 1.** Bioequivalence analysis results of oseltamivir

**Additional file 2.** Summary table of adverse events

**Additional file 3.** Summary of all treatment emergent adverse events(TEAEs) based on system organ class(SOC), preferred terminology(PT)

## Data Availability

A signed confidential document, dataset generated and/or analyzed during the current study is not publicly available, but can be obtained directly from the author upon reasonable request. To obtain data for this study, please contact Ms. Ying Wang or Mr. Bangzhong Tang.

## Abbreviations

AE: Adverse event
AUC_0-t_: Area under the plasma concentration–time curve from time 0 to last time of quantifiable concentration
AUC_0-∞_: Area under the plasma concentration–time curve from time zero extrapolated to infinite time
%AUC _ex_: Percentage of AUC0-∞ obtained by extrapolation
BMI: Body mass index
BQL: Below the limit of quantification
C _max_: Maximum concentration
CL/F: Apparent clearance
CTCAE: Common Terminology Criteria for Adverse Events
%CV: Coefficient of variation
%CV_W_: Within-subject coefficient of variation
GLSM: Geometric least squares mean
h: Hour
LC–MS/MS: Liquid chromate grapy tandem mass spectrometry
Max: Maximum
min: Minute
PT: Preferred term
PK: Pharmacokinetics
SAE: Serious adverse event
SAS: Statistical analysis software
SD: Standard deviation
SOC: System organ class
t_1/2_: Elimination half life
T _max_: Time to reach maximum concentration
V _d_/F: Apparent volume of distribution
λ _z_: Elimination rate constant
